# Supplementary material for: Patterns of orthopedic and trauma admissions to a tertiary teaching and referral health facility in Kenya: Chart review
Source: PLOS Glob Public Health. 2023 May 17;3(5):e0001425. doi: 10.1371/journal.pgph.0001425 (PMC10191292; doi:10.1371/journal.pgph.0001425)
Supplement: Attachment — Submitted filename: Response to Reviewers.docx [file pgph.0001425.s003.docx]

**LETTER OF REBUTAL – 24^TH^ MARCH, 2023**

| Comments | Authors response |
| --- | --- |
| Journal Requirements: |  |
| 1. Please send a completed 'Competing Interests' statement, including any COIs declared by your co-authors. If you have no competing interests to declare, please state "The authors have declared that no competing interests exist". Otherwise please declare all competing interests beginning with the statement "I have read the journal's policy and the authors of this manuscript have the following competing interests:" | Done |
| 2. Please amend your detailed Financial Disclosure statement. This is published with the article. It must therefore be completed in full sentences and contain the exact wording you wish to be published.  a. State the initials, alongside each funding source, of each author to receive each grant. b. If any authors received a salary from any of your funders, please state which authors and which funders.  If you did not receive any funding for this study, please simply state: “The authors received no specific funding for this work.” | Done |
| 3. Please provide separate figure files in .tif or .eps format only and remove any figures embedded in your manuscript file. Please also ensure that all files are under our size limit of 10MB. | Done |
| 4. In the online submission form, you indicated that "We have full access of the original raw data and will share the data on request on short notice". All PLOS journals now require all data underlying the findings described in their manuscript to be freely available to other researchers, either 1. In a public repository, 2. Within the manuscript itself, or 3. Uploaded as supplementary information. This policy applies to all data except where public deposition would breach compliance with the protocol approved by your research ethics board. If your data cannot be made publicly available for ethical or legal reasons (e.g., public availability would compromise patient privacy), please explain your reasons by return email and your exemption request will be escalated to the editor for approval. Your exemption request will be handled independently and will not hold up the peer review process, but will need to be resolved should your manuscript be accepted for publication. One of the Editorial team will then be in touch if there are any issues. | Is uploaded as a supplementary file. |
| 5. Fig 1: please (a) provide a direct link to the base layer of the map (i.e., the country or region border shape) and ensure this is also included in the figure legend; and (b) provide a link to the terms of use / license information for the base layer image or shapefile. We cannot publish proprietary or copyrighted maps (e.g. Google Maps, Mapquest) and the terms of use for your map base layer must be compatible with our CC-BY 4.0 license.  Note: if you created the map in a software program like R or ArcGIS, please locate and indicate the source of the basemap shapefile onto which data has been plotted.  If your map was obtained from a copyrighted source please amend the figure so that the base map used is from an openly available source. Alternatively, please provide explicit written permission from the copyright holder granting you the right to publish the material under our CC-BY 4.0 license.  Please note that the following CC BY licenses are compatible with PLOS license: CC BY 4.0, CC BY 2.0 and CC BY 3.0, meanwhile such licenses as CC BY-ND 3.0 and others are not compatible due to additional restrictions.  If you are unsure whether you can use a map or not, please do reach out and we will be able to help you. The following websites are good examples of where you can source open access or public domain maps:  * U.S. Geological Survey (USGS) - All maps are in the public domain. ([http://www.usgs.gov](http://www.usgs.gov/))  * PlaniGlobe - All maps are published under a Creative Commons license so please cite “PlaniGlobe, [http://www.planiglobe.com](http://www.planiglobe.com/), CC BY 2.0” in the image credit after the caption. (<http://www.planiglobe.com/?lang=enl>)  * Natural Earth - All maps are public domain. (<http://www.naturalearthdata.com/about/terms-of-use/>) | Done. See page 16  a. The link to the county layers is the DIVA-GIS website (<https://www.diva-gis.org/datadown>) in shape file format. Note that this was the only shapefile used for map production.  Besides, no base layer was used. This data is freely available.  b. The map was produced in QGIS version 3.24 (QGIS Development Team 2014) which is also an open-source software package. |
| Reviewers’ comments |  |
| Reviewer #1: Congratulations on an excellent work done. I recommend adding a Recommendations paragraph for policy advocacy of Trauma guidelines  Perhaps think of extending the study to include more African countries or setup a Trauma registry to include more African countries. | Done – see page 22 of the unmarked manuscript  Well noted. This will be considered in the follow up study |
| Reviewer #2: This manuscript has some new observation and is of locoregional interest. However, there are some grammatical and syntax errors and repetition of of words in the text and references. It may be checked by an native English speaker.  The references are not uniform in style and missing dates in some. These needs to be addressed. | Done  Done and Endnote software used to generate automatic references – see pages 23 - 24 |
| Reviewer #3: This manuscript describes the presentation of trauma patients to Kenyatta National Hospital. I commend the authors for collecting and presenting a large volume of important data, however, I believe the manuscript needs significant work to realise its full potential.  1. The authors give a lot of data about presentation, but there is no data on what the actual injuries were, treatments, or outcomes. Even if the focus of the study is on presentation, readers would benefit from at least brief detail on these other issues, in order to contextualise.  2. I understand that there is little epidemiological data for these patients in Kenya, but with a purely descriptive study I am left wondering what the implications of this data are? The authors have not drawn out any clear recommendations for priorities or potential solutions to improve patient care. I wonder if they might better placed to do this if they form a clear hypothesis which they can address with their data. For example, could they compare self-referred versus clinician-referred patients in terms of presentation and outcome to inform how care pathways could be improved?  3. There is a lot of repetition of information between the results text, tables, and discussion - please try to avoid repetition so that you can reduce the overall length of the manuscript to 2500 words or so. A shorter, more focussed manuscript will mean readers take away the key points you want them to have. Some tables could be moved to a supplementary file, if needed.  4. Since this manuscript is focused on a single hospital, it would be helpful to give some context by describing the hospital, available resources, and services it offers.  6. Please include a completed STROBE checklist. | Done. See page 15 for type of injuries. However, we did not collect variables on treatment given and outcome for this study.  Done. See page 15 on type of injuries comparing walk-in patients and facility referred patients. Also see page 22 on recommendations sections  Noted and revised/edited across the manuscript.  Done. See page 3 and page 4 of the unmarked manuscript.  Done – filed as a supplementary file |
| Reviewer #4: I would like to thank you for the opportunity to review this interesting manuscript and congratulate the team on their work. Regarding the method, instead of using a full calendar year, why was the study period only 11 months? It would be very helpful to compare the results across years to arrive at a robust conclusion where data are available. It would be interesting to assess the appropriateness of these emergency visits that were treated in relation to the level of care to see if a better triage and referral system may improve the health system's performance.  Additional literature research is needed to better frame the research question, and further discussion is needed to connect the findings with health system challenges.  Lastly, the data supplied in the paper was not independent of statistical software. | The study ended up covering 11 months since the desired sample for the study was attained  The study did not collect variables that assess the appropriateness of emergency visits in relation to the level of are but we did collect the types of orthopedic and trauma conditions admitted and we have presented that in table 8 page 15 of the unmarked manuscript.  Done. Discussion section reviewed to reflect health system challenges and these are also captured in the recommendation section.  The raw data has been attached as supplementary file for review. SPSS version 21 was used for analysis. |
